# Outcomes in Radiotherapy-Treated Patients With Cancer During the COVID-19 Outbreak in Wuhan, China

**DOI:** 10.1001/jamaoncol.2020.2783

**Published:** 2020-07-30

**Authors:** Conghua Xie, Xiaoyong Wang, Hui Liu, Zhirong Bao, Jing Yu, Yahua Zhong, Melvin L. K. Chua

**Affiliations:** 1 Department of Radiation and Medical Oncology, Zhongnan Hospital of Wuhan University, Wuhan, China; 2 Hubei Key Laboratory of Tumor Biological Behaviors, Zhongnan Hospital of Wuhan University, Wuhan, China; 3 Hubei Cancer Clinical Study Center, Zhongnan Hospital of Wuhan University, Wuhan, China; 4 Division of Radiation Oncology, Division of Medical Sciences, National Cancer Centre Singapore, Singapore; 5 Oncology Academic Clinical Programme, Duke–National University of Singapore Medical School, National University of Singapore, Singapore

## Abstract

This case series evaluated the delivery of radiotherapy in 209 patients with cancer during the COVID-19 outbreak in Wuhan, China.

Several health care services have been affected by the novel coronavirus disease 2019 (COVID-19) pandemic. A delay in diagnosis and treatment can be detrimental to patients with cancer.^1,2^ However, patients with cancer are also at risk for COVID-19 because of immunosuppressive treatments and recurrent visits to the hospital.^3^ In this article, we report preliminary outcomes in 209 patients who underwent radiotherapy at the Zhongnan Hospital of Wuhan University (ZHWU) during the COVID-19 outbreak in the city of Wuhan, China.

## Methods

All patients who were treated at the Department of Medical and Radiation Oncology, ZHWU, from January 20 to March 5, 2020, were included. Public health measures implemented during the study period included city lockdown (January 23, 2020), *cordon sanitaire*, traffic restriction, social distancing, and home confinement. We analyzed patient demographics as well as clinical and treatment parameters. Survival status of all patients was updated as of March 12, 2020.

This study was approved by the ZHWU institutional review board (No. 2020041) with waiver of informed consent for the use of aggregated, anonymized patient data.

## Results

The Table summarizes the clinical characteristics of 209 patients and their treatment details. Median (interquartile range) age of the patients was 55 (48-64) years; 104 patients (49.8%) were men, and 105 (50.2%) were women. Most patients had thoracic cancer (n = 80 [38.3%], including lung, breast, and esophageal cancers), head and neck cancer (n = 53 [25.4%]), or gastrointestinal or gynecological cancer (n = 54 [25.8%]). Of the patients, 99 (47.4%) received adjuvant radiotherapy, whereas 57 (27.3%) and 53 (25.3%) underwent radical and palliative radiotherapy, respectively; 67 patients (32.1%) received concurrent chemoradiotherapy. All patients had already begun treatment prior to the study start date.

**Table.** Clinical and Treatment Characteristics of the Study Patients

Characteristic	No. (%)
**Clinical details**	
Sex	
Male	104 (49.8)
Female	105 (50.2)
Age, median (IQR), y	55 (48-64)
Cancer diagnosis	
Head and neck	53 (25.4)
Thoracic^a^	80 (38.3)
Lower gastrointestinal and gynecological	54 (25.8)
Others	22 (10.5)
Hospitalized	172 (82.3)
Outpatient	37 (17.7)
**Treatment details**	
Radiotherapy alone	142 (67.9)
Concurrent chemotherapy and RT	67 (32.1)
RT details	
Radical	57 (27.3)
Adjuvant	99 (47.4)
Palliative	53 (25.3)
RT regimens	
Conventional	186 (89.0)
Hypofractionation	23 (11.0)
Phase of RT at the start of study period	
Week 1-2	191 (91.4)
Week 3-4	11 (5.3)
Week 5-7	7 (3.3)
Treatment interruption after lockdown, No. of patients	
RT interruption^b^	112 (53.6)
Chemotherapy interruption^c^	62
No. of RT sessions per day, mean (range)	
Before lockdown	188 (160-209)
After lockdown	12 (2-66)

Abbreviations: IQR, interquartile range; RT, radiotherapy.

^a^ Includes lung, breast, and esophageal cancer patients.

^b^ Lockdown of Wuhan city occurred on January 23, 2020.

^c^ 58 discontinued due to lockdown; 4 discontinued due to physician decision.

Unfortunately, 112 patients (53.6%) were unable to return for radiotherapy after the lockdown. Among the 67 patients receiving chemoradiotherapy, 3 (4.5%) had completed treatment and 62 (92.5%) discontinued treatment (58 could not return, and 4 discontinued by the physician’s choice); only 2 patients (3.0%) resumed chemoradiotherapy. Before the lockdown, the mean (range) number of patients per day was 188 (160-209). However, these numbers dropped sharply after the date of lockdown and declined with each subsequent week (mean [range] number of patients per day, 12 [2-66]) (Figure).

**Figure.**
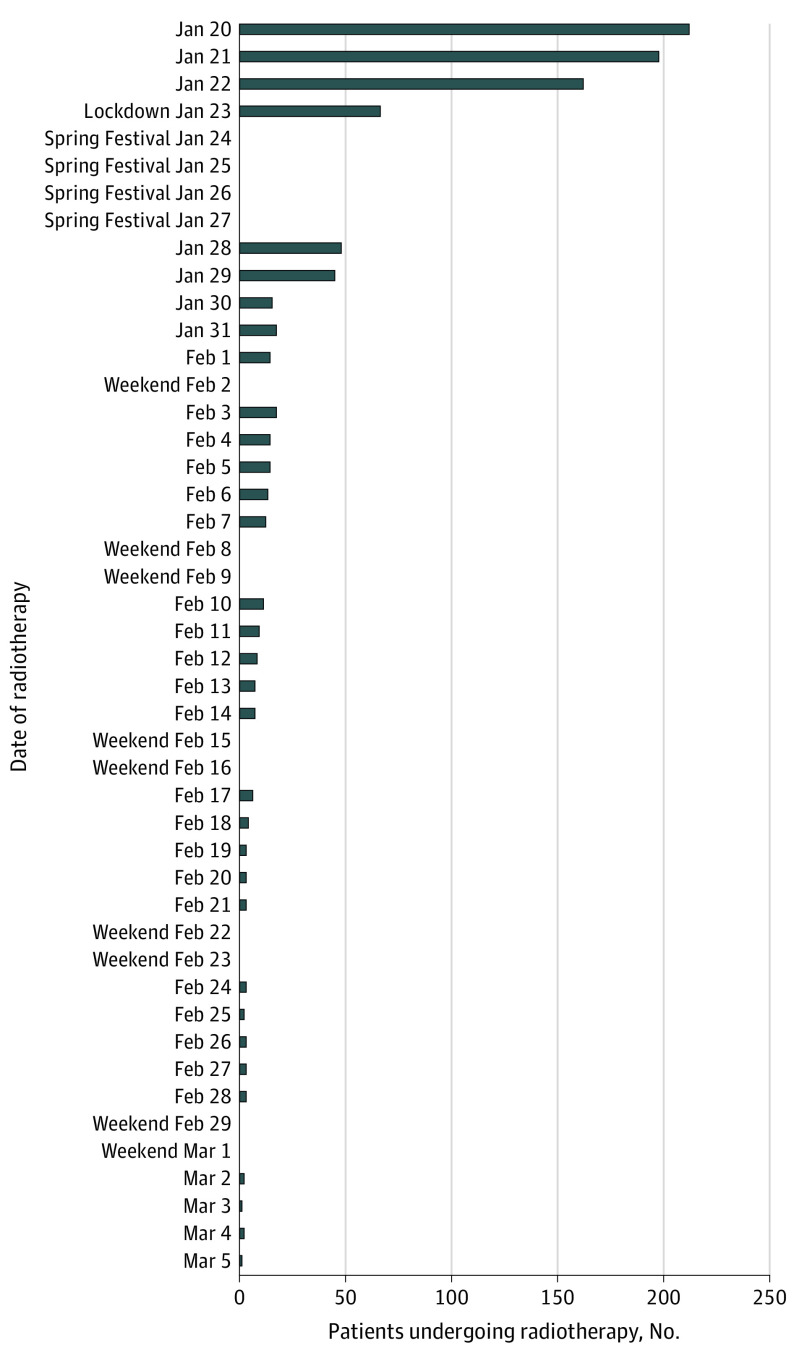
Radiotherapy Caseload per Day During the Coronavirus Disease 2019 Outbreak Influence of public health measures on the daily number of patients undergoing radiotherapy at the Zhongnan Hospital of Wuhan University.

We recorded only 1 case (0.5%) of confirmed severe acute respiratory syndrome coronavirus 2 infection during the study period. Although 70 patients (33.5%) had a history of contact with this patient, none of them developed clinical symptoms of COVID-19. Of these 70 patients, 52 (74.2%) were unable to resume radiotherapy after the lockdown, while 18 (25.8%) continued radiotherapy without delay. All patients were alive as of March 12, 2020.

## Discussion

To date, more than 10 000 000 humans have been diagnosed as having COVID-19. This disease is highly infectious, since both asymptomatic and symptomatic individuals can transmit the virus.^4,5^ Extensive public health measures that are focused on physical distancing and tight containment have been implemented. In the city of Wuhan, China, such measures were effective in limiting virus transmission and reducing daily new COVID-19 cases across all age groups.^6^ However, there are concerns that these public health measures will affect the delivery of other health care services.

In this article, we share our experience with the COVID-19 lockdown and the delivery of radiotherapy in patients with cancer at ZHWU in Wuhan, China. Caseloads were substantially reduced (a 10-fold drop after lockdown). More than half of the patients in this case series were unable to return to the city for treatment, which is a consequence of the massive human migration (*Chunyun*) for the Spring Festival that preceded the lockdown. Additionally, physicians were conservative in resuming chemoradiotherapy. Long-term follow-up data may reveal detrimental ramifications of treatment interruption on the survival of these patients with cancer.
